# Effect of Microgravity on Fungistatic Activity of an α-Aminophosphonate Chitosan Derivative against *Aspergillus niger*

**DOI:** 10.1371/journal.pone.0139303

**Published:** 2015-10-15

**Authors:** Kesavan Devarayan, Yesupatham Sathishkumar, Yang Soo Lee, Byoung-Suhk Kim

**Affiliations:** 1 Department of Organic Materials & Fiber Engineering, Chonbuk National University, 567 Baekje-daero, Deokjin-gu, Jeonju-si, Jeollabuk-do 54896, Republic of Korea; 2 Department of BIN Convergence Technology, Chonbuk National University, 567 Baekje-daero, Deokjin-gu, Jeonju-si, Jeollabuk-do 54896, Republic of Korea; 3 Department of Forest Science and Technology, College of Agriculture and Life Sciences, Chonbuk National University, 567 Baekje-daero, Deokjin-gu, Jeonju-si, Jeollabuk-do 54896, Republic of Korea; 4 Department of Basic Sciences, College of Fisheries Engineering, Tamil Nadu Fisheries University, Nagapattinam, India; Woosuk University, REPUBLIC OF KOREA

## Abstract

Biocontamination within the international space station is ever increasing mainly due to human activity. Control of microorganisms such as fungi and bacteria are important to maintain the well-being of the astronauts during long-term stay in space since the immune functions of astronauts are compromised under microgravity. For the first time control of the growth of an opportunistic pathogen, *Aspergillus niger*, under microgravity is studied in the presence of α-aminophosphonate chitosan. A low-shear modelled microgravity was used to mimic the conditions similar to space. The results indicated that the α-aminophosphonate chitosan inhibited the fungal growth significantly under microgravity. In addition, the inhibition mechanism of the modified chitosan was studied by UV-Visible spectroscopy and cyclic voltammetry. This work highlighted the role of a bio-based chitosan derivative to act as a disinfectant in space stations to remove fungal contaminants.

## Introduction

Exploration of space by manned missions has a main goal of being able to prolong the stay in low earth orbit as well as on extraterrestrial planets. The health and well-being of the astronauts and the crew are most important for long-term stay in space. It is inevitable that the population of microorganisms such as bacteria and fungi are ever increasing in space, mainly due to human activity [1]. Although most of the microorganisms are not a threat to humans with a healthy immune system, it was reported that the immune functions of the astronauts are compromised under microgravity. This is mainly due to the unique circumstances, such as microgravity, radiation, hypobaric, and restricted hygienic practices [2–4].

Previous studies on bacteria, for instance, *Salmonella enterica* and *Salmonella typhimurium*, under simulated microgravity conditions revealed that the changes in gravity factor can induces genotypic changes in these organisms [5–7]. Further, the strains grown under microgravity conditions exhibited increased virulence and increased resistance to environmental stresses such as thermal, acid, and osmotic stresses, leading to antibiotic resistance.

Besides bacteria and viruses, another important group of pathogens is fungi. Recently, the experiments performed in space flight or modelled microgravity conditions revealed that the microgravity induces changes in the hyphal trajectory and intracellular morphological changes in the fungi such as *Ulocladium chartarum*, *Penicillium chrysogenum*, and *Aspergillus niger* (*A*. *niger*) [8, 9]. Due to the immune dysfunction in astronauts under microgravity, *A*. *niger* can emerge as an opportunistic pathogen and cause a direct threat to the health of crew members. The fungal growth on organic substrates is another notable problem caused by fungi. In order to grow and multiply, fungi can utilize any organic substances, which could be the working parts of space stations such as rubber seals of windows, component of space suits, insulations on cables, tubings, and other communication devices [10].

Among the fungi found in International Space Station (ISS), *A*. *niger*, an opportunistic pathogen, was predominant as a biocontaminant. It was reported that the dust sample collected from ISS contained *A*. *niger* at higher levels than its presence in the homes in U.S. [10, 11]. However, the knowledge on survival and growth of fungi in response to any antifungal agents in microgravity is limited, and the corresponding changes to infectious diseases are also unclear.

Generally, polycations are used as antimicrobials for clinical and domestic purposes in recent decades. Among them, poly quaternary amines are promising as antifungal agents [12]. Chitosan, a polycationic polymer of glucosamine, exhibited significant antifungal activity against several organisms including *A*. *niger* [13, 14]. A strategy to chemically combine small active molecules with chitosan by means of alkylation, acylation, or by quaternarization, can provide a synergistic effect towards its antifungal activity [15, 16]. α-amino phosphonates are an important group of compounds, which are structural analogs of naturally-occurring amino acids present in peptides. α-amino phosphonates were reported to act as antiviral, antifungal, antibacterial, and in some cases antitumour agents [17–21]. In view of enhancing the fungicidal efficacy of chitosan, we designed a simple synthetic strategy to synthesize a chitosan derivative containing α-aminophosphonate and quaternary amine groups.

Eventhough, *A*. *niger* is being found to be a major biocontaminant in spaceships, there is to date no report for the evaluation of the response of the *A*. *niger* against any antifungal agent under microgravity. Thus, the present study has been undertaken with the following objectives: (i) facile synthesis of chitosan derivative containing α-amino phosphonate and quaternary ammonium group, (ii) evaluation of the fungistatic activity of the new chitosan derivative under microgravity, and (iii) examining the survival of *A*. *niger* grown under both normal and microgravity in presence of the chitosan derivative. In order to imitate the gravity factor similar to the space, a high aspect ratio vessel equipped with a specialized membrane was employed to develop a low-shear modelled microgravity environment. This study would be the first to initiate the examination of fungicidal potency of chitosan derivative and first to observe the survival of the fungi against a fungistatic agent under microgravity.

## Materials and Methods

### Materials

Low molecular weight acid-soluble chitosan (manufacturer’s specification, *M*_*w*_ = 50000 g/mol; DDA = 80%), dimethylamino benzaldehyde (DMAB), and dibutyl phosphite were purchased from Sigma-Aldrich, USA and used without further purification unless otherwise stated.

#### Synthesis of N-(p-dimethylaminobenzyl) chitosan

At first, the chitosan-Schiff base was prepared from chitosan and DMAB via a reductive amination reaction. Chitosan (2.0 g) and DMAB (1.0 eq.mol) were mixed in 30 mL of ethanol and refluxed for 10 h. Then dimethyl phosphite (1.2 eq.mol/NH_2_) was added dropwise to the reaction mixture and stirred for 10 h at 90°C. The reaction mixture was cooled down to 40°C and then filtered. The precipitate was washed with distilled water and ethanol and then dried under vacuum for 24 h to obtain *N*-(p-dimethylaminobenzyl)-dimethyl-α-aminophosphonate chitosan [DMAB(P)-Ch], yellow powder (yield: 65.1%). ^31^P NMR (1% CD_3_COOD/D_2_O, 400 MHz, δ): 8.2 ppm. FT-IR (KBr): 3200–3550 cm^-1^ (ν_O-H+N-H_), 2976 cm^-1^ (asym. ν_C-H_, -CH_2_), 2933 cm^-1^ (sym. ν_C-H_, -CH_3_), 1662 cm^-1^ (ν_C = O_, amide I), 1603 cm^-1^ (N-H def, amide-II & NH^+^), 1535 cm^-1^ (ν_N-H_ phenyl), 1477 cm^-1^ (ν_C-H_, -CH_2_-CH_3_ phosphonate), 1255 cm^-1^ (ν_P = O_, phosphonate), 825 cm^-1^ (phenyl), 716 cm^-1^ (ν_P-C_). Elemental composition: C, 43.98%; N, 21.52%; O, 27.14%; P, 7.37%.

#### Characterization of chitosan derivative

All the samples were characterized by means of FT-IR spectroscopy (Perkin-Elmer) and UV-visible spectroscopy (Shimadzu UV-1800, Tokyo, Japan). ^31^P NMR was measured using a JEOL A-400 NMR Spectrometer. Elemental composition was performed using a JEOL JSM-5900 scanning electron microscope equipped with energy dispersive spectrum (SEM-EDS).

#### Fungal strain

*Aspergillus niger* (*A*. *niger*) (KACC 42589) purchased from Korean Agricultural Culture Collection (Suwon, South Korea). Fungal culture was maintained on potato dextrose agar (BD Difco, Sparks, MD) supplemented with streptomycin (100 mg/L).

#### Low-shear modelled microgravity

In this study, microgravity was simulated in a rotatory cell culture system (RCCS-1) that was purchased from Synthecon, Incorporated (Houston, Texas 77054 USA) with Autoclavable High Aspect Ratio Vessel (HARV) of 50 mL capacity. Potato dextrose broth (PDB) (BD Difco, Sparks, MD) was prepared according to manufacturer’s instruction (2.4 g/100 mL). At first, HARV was filled with 49 mL of PDB with streptomycin and 1 mL of seed culture (~10^6^ spores/mL) was added. Then HARV was rotated in the horizontal and vertical axis at 25 rpm to obtain the microgravity and normal gravity condition, respectively. At horizontal axis rotation, the gravitational vectors were randomized over the surface of the cell and thus resulting in microgravity environment with an overall-time-averaged gravitational vector of 10^−2^ g [22]. The HARV was placed inside the humidity chamber with 90% humidity and maintained at 25°C to avoid formation of air bubbles that might cause turbulence and affecting the fluid dynamics during experiment due to evaporation of the medium. The germination rates were observed for 24 h with 4 h interval time using a light microscope (Olympus BX41TF, Tokyo, Japan). 200 spores (germinated and non-germinated) were counted. A spore was considered to be germinated, when the length of it's germinal tube reached one half of the diameter of the spore [23]. Experiments were performed in triplicates and repeated. Similar to the control, experiments were performed with Ch, DMAB, and DMAB(P)-Ch.

#### Radial hyphal growth bioassay

PDB was prepared as described above. Agar 1% (w/v) and Chitosan derivative to a final concentration of 500 μg/mL was added. The solution was autoclaved for 15 min at 121°C and 15 psi. After cooling around 70˚C the solution 20 mL was poured into a sterile petri dish (9 cm diameter) resulting in 4 mm deep agar and allowed to solidify. Margin of the 7 day old culture of *A*. *niger* was touched with a needle and pointwise puncture inoculation was carried out by deposition of the inoculum to the center of the plate roughly 2 mm deep and plates were incubated at 25°C. Colony diameter was measured along the line passing through the center of the colony using a transparent ruler. An average of 5 readings was taken for the calculation. The determination of radial extension growth of the colony was measured at 2 days and 5 days and compared with the growth in control media. The fungal inhibition efficiency (*FIE%*) was calculated as given below in Eq 1.

$$FIE\%=\frac{\left(C_{b}-C_{t}\right)}{\left(C_{b}\right)}\times 100$$ (1)

Where *C*_*b*_ and *C*_*t*_ are diameters of the fungal colony in absence and presence of chitosan derivative, respectively.

#### Qualitative analysis of secondary metabolites

10 mL of PDB with varying concentrations of DMAB(P)-Ch of 500 μg/mL, 50 μg/mL, and 0 μg/mL was used for the assay. Approximately, 1 mL of stock solution(containing ~10^6^ spores/mL) was added to each tube and incubated at 25°C for 2 weeks. For cyclic voltammetry measurements, 1 mL of the test solution was diluted with 74 mL of 100 mM KCl solution. The cyclic voltammetry measurements were carried out using VersaStat4 electrochemical analyzer at the range of -0.4 to 0.9 V (vs Ag/AgCl) at the sweep rate of 500 mV/s. The electrochemical cell consisted of a platinum wire as counter electrode, an Ag/AgCl as the reference electrode, and a glassy carbon as working electrode. Further, the samples were also analyzed by UV-Visible spectroscopy.

#### Scanning electron microscopy

Afte exposing to DMAB(P)-Ch, the fungal hyphae were briefly washed in sterile water. Primary fixation of fungal hyphae was done with 2.5% glutaraldehyde and 2% paraformaldehyde buffered with 0.01 M phosphate buffered saline for 4 h at 4°C. Post fixation was done with 1% osmium tetroxide (Tedpella, Redding, CA) in 0.05 M PBS pH-7.2 for 2 h. Samples briefly washed with distilled water at room temperature and kept at 4°C with 0.5% uranyl acetate (EMS, Hatfield, USA). Samples were dehydrated with graded ethanol series 30, 40, 50, 60, 70, 80, 90% and three changes of 100% ethanol 10 min for each alcohol dilution. The samples were kept in a vacuum desiccator until they were completely dry. Samples were placed on a stub coated with carbon electron conductive tape. Sputter coated with platinum for 120 s and examined under scanning electron microscope (JEOL JSM-5900, Japan).

## Results and Discussion

### Synthesis and characterization of N-(p-dimethylaminobenzyl)-dimethyl-α-aminophosphonate chitosan

**Fig 1:**
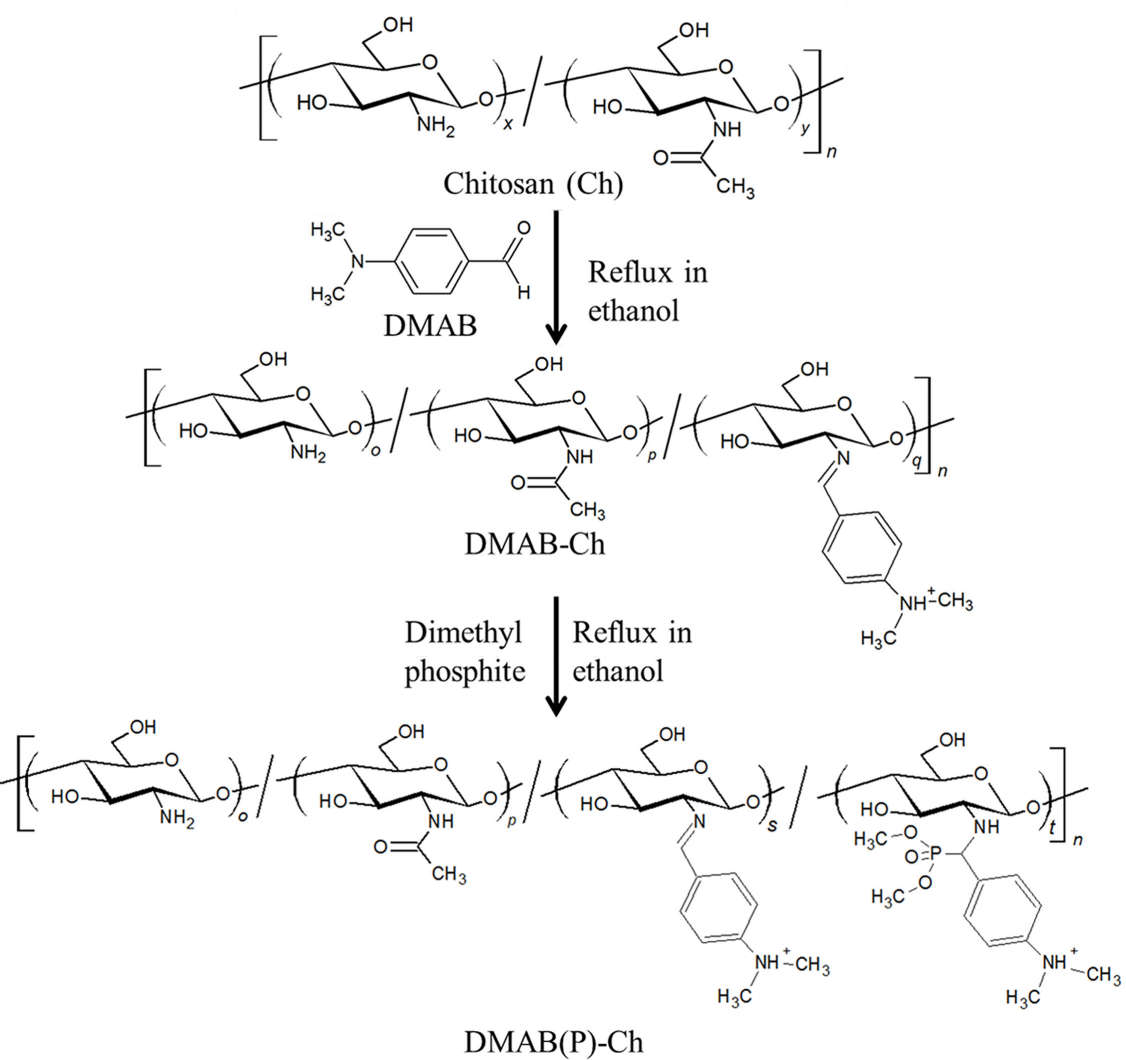
Schematic representation of synthesis of α-aminophosphonate chitosan derivative.

**Fig 2:**
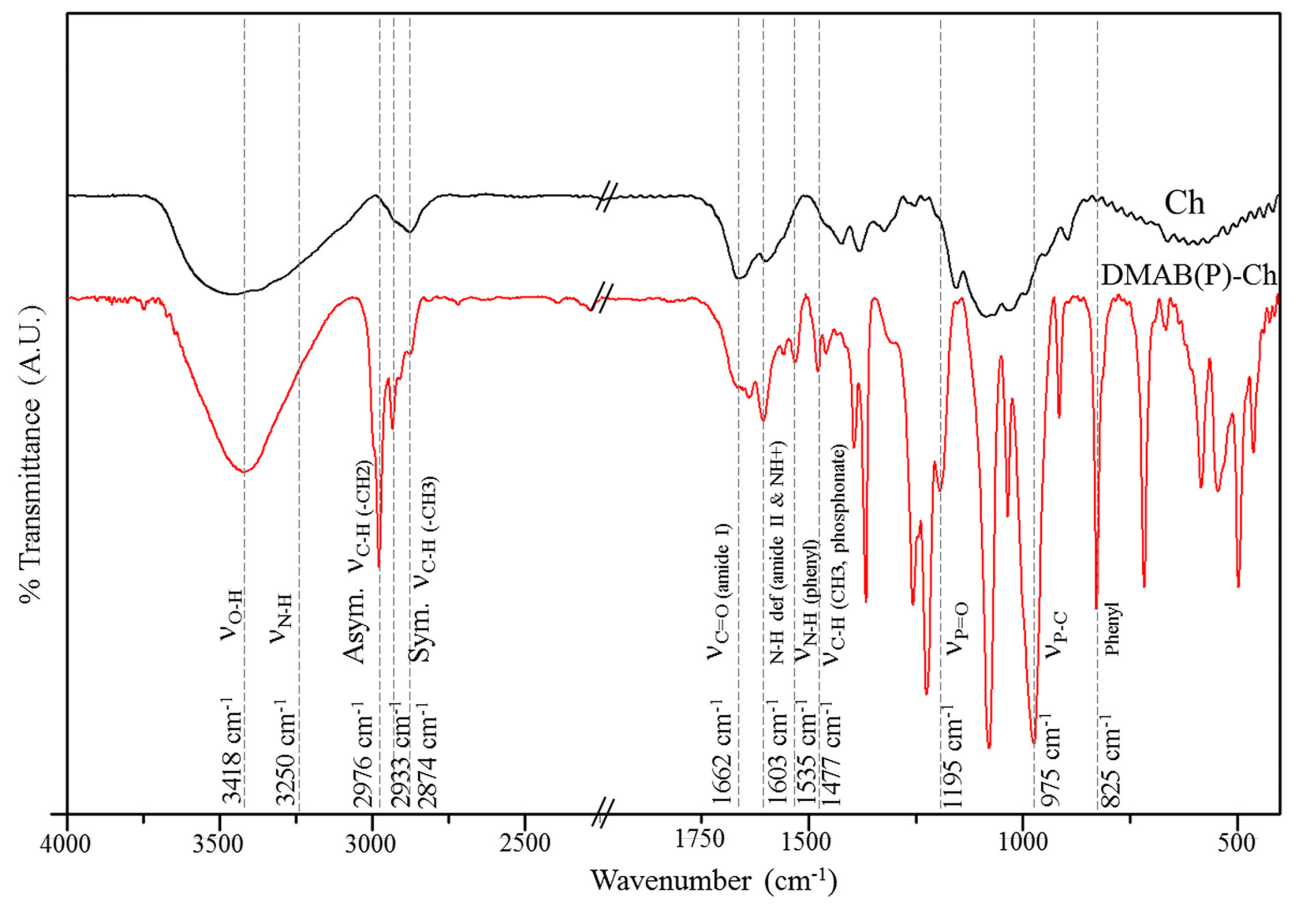
FT-IR spectroscopy of Ch and DMAB(P)-Ch.

*α*-aminophosphonate derivative of chitosan was synthesized by a catalyst-free Pudovik reaction [24, 25]. At first the Schiff base was prepared from DMAB and Ch to form DMAB-Ch. Subsequent addition of dibutyl phosphite to DMAB-Ch at 90°C yielded the α-aminophosphonate DMAB(P)-Ch (Fig 1). Fig 2 shows the FT-IR spectra of Ch and DMAB(P)-Ch. The broad band from 3200 to 3500 cm^-1^ was attributed to the stretching vibrations of–OH and–NH. The peak at 2976 and 2933 cm^-1^ were due to C-H asymmetric and symmetric stretching vibrations of DMAB(P)-Ch, respectively. The amide-I and amide-II bands were observed at 1662 and 1603 cm^-1^, respectively. The increase in the intensity of the amide-II band is possibly due to the overlapped vibrations of NH^+^ with amide II. Significant peaks for the stretching vibrations of P = O, P-C were observed at 1195 and 975 cm^-1^, indicated the presence of phosphonate group in DMAB(P)-Ch.

**Fig 3:**
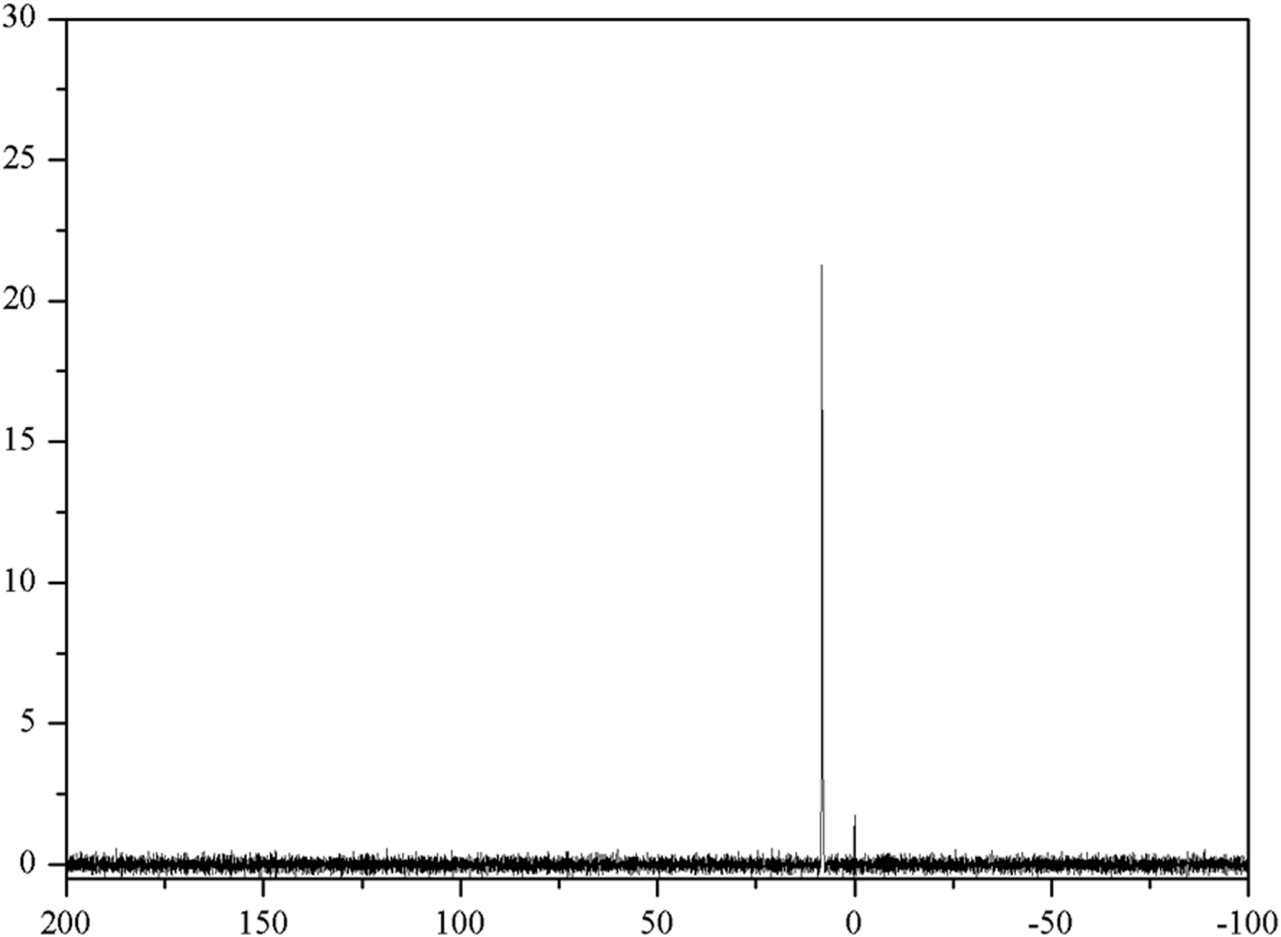
^31^P NMR spectroscopy of DMAB(P)-Ch.

**Fig 4:**
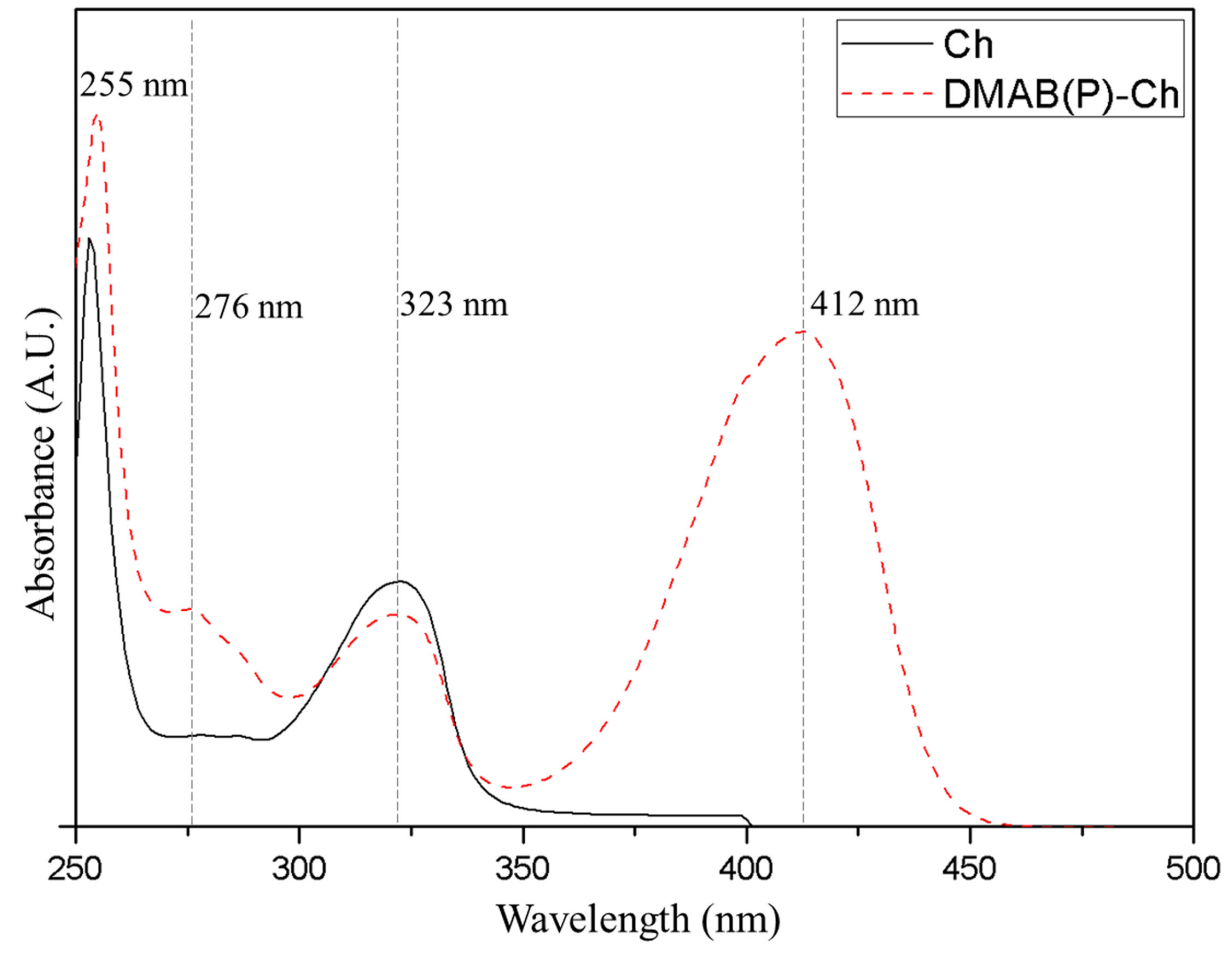
UV-Vis spectral data of Ch and DMAB(P)-Ch.

The presence of phosphonate in DMAB(P)-Ch was further confirmed by ^31^P NMR spectroscopy. The ^31^P NMR signal observed at 8.2 ppm in Fig 3, confirms the successful synthesize of DMAB(P)-Ch. Fig 4 shows the UV-Visible spectra of Ch and DMAB(P)-Ch. The Ch exhibited absorption bands at 255 and 323 nm corresponding the n→π* transitions. In addition these bands, a new band at 276 nm was observed for DMAB(P)-Ch, which was attributed to π→π* of the phenyl group. Further, intermolecular charge transfer transition observed at 412 nm indicated possible formation of quaternary ammonium structure. The elemental analysis results revealed that the synthesized α-aminophosphonate DMAB(P)-Ch contained about 7.37% of phosphonate. These results confirmed the successful synthesize of an α-aminophosphonate chitosan derivative containing quaternary ammonium group.

### Fungistatic Effect of DMAB(P)-Ch

**Fig 5:**
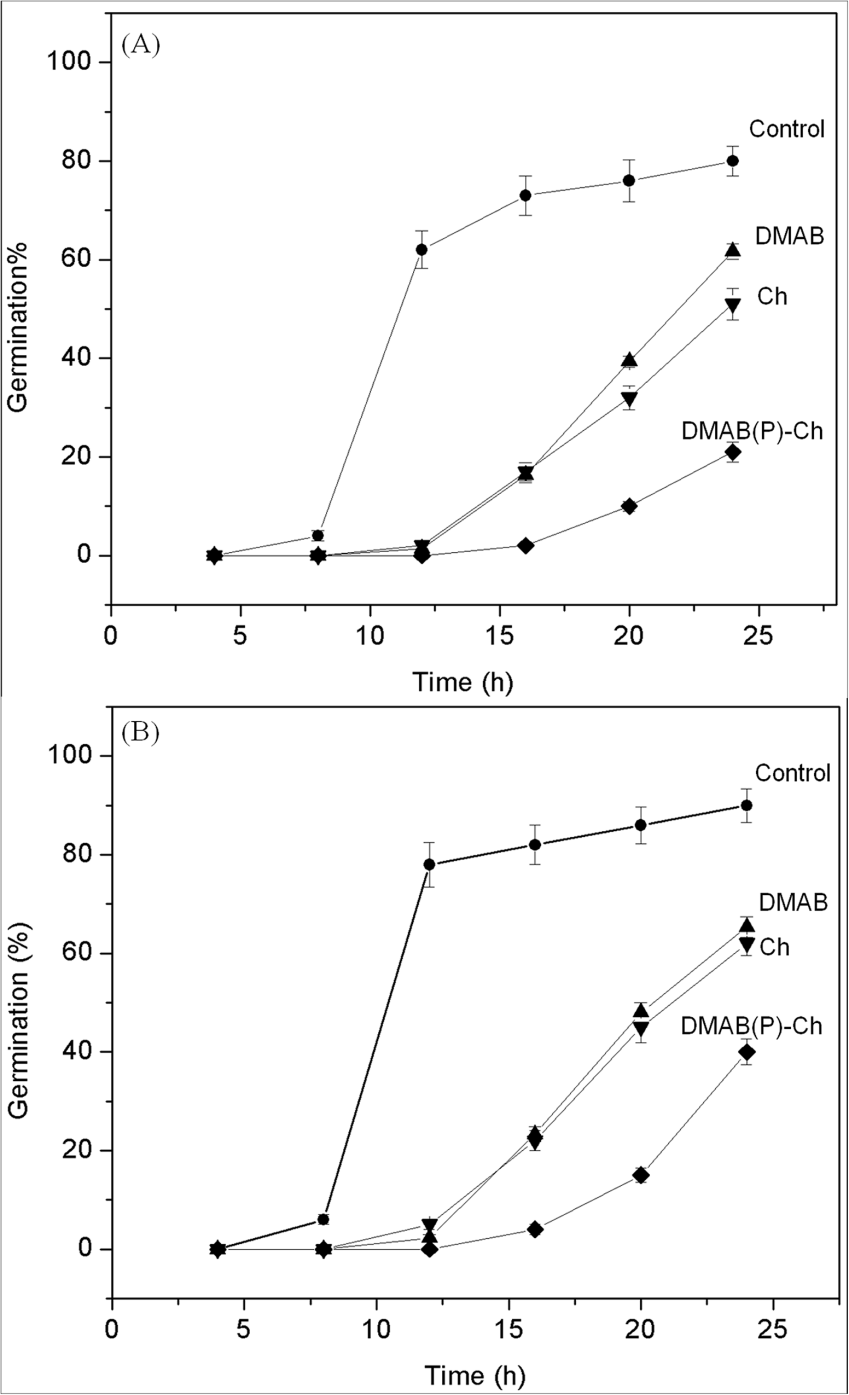
*A*. *niger* spore germination growth curve (A) normal gravity and (B) microgravity.

Spore germination was observed in the presence and absence of Ch, DMAB, and DMAB(P)-Ch under both normal and microgravity. Under normal gravity conditions, about 80% of spores were germinated after 24 h (Fig 5A) in control. Meanwhile, 51 and 62% of spores were germinated in the presence of Ch and DMAB, respectively. Whereas, only 21% of spores were able to germinate in the presence of DMAB(P)-Ch. It should be noted that in control experiments, spores started to germinate at 8 h. On the other hand, in presence of Ch and DMAB(P)-Ch, germination started at 12 and 16 h, respectively. This could be due to the chelation of minerals, in particular Ca^2+^ ions or nutrients with DMAB(P)-Ch. It was reported that calcium is one of the essential minerals for growth and spore germination [26]. The spore germination results indicated that the modified chitisan derivative, DMAB(P)-Ch has showed significant synergistic effect.

After confirming the inhibitory effect of DMAB(P)-Ch under normal gravity, the efficacy of the chitosan derivative was tested under microgravity. Interstingly, the spore germination pattern similar to normal gravity was observed for microgravity experiments (Fig 5B). It is noteworthy that DMAB(P)-Ch is effective under microgravity against *A*. *niger*.

### Radial Hyphal Assay

The fungistatic effect of DMAB(P)-Ch under normal gravity gradually decreased with the incubation time. After 2 days of inoculation, the maximal percentage of radial growth inhibition at the concentration of 50 μg/mL was approximately 65%, and after 5 days FIE% was 19% (S1 Fig & Table 1). However, the FIE% drastically increased to 76% and 38% for 2 days and 5 days of incubation, respectively, with the concentration of DMAB(P)-Ch being 500 μg/mL as shown in Table 1. Same pattern was observed in the liquid medium while measuring the fresh weight and dry weight of the biomass grown under normal gravity conditions. Fresh weight of the mycelium grown under normal gravity after 2 days both in the presence and absence of chitosan derivative were 4.5 g and 2.5 g, respectively (Table 2). It is noteworthy that about 0.5 g of fresh weight increased for mycelium grown under microgravity. This increase could be due to the enhanced availability of nutrients in the medium to the growing fungus under weightless environment and partially due to the gene expression changes in *A*. *niger* [8].

**Table 1:** Comparative antifungal index for normal gravity *A. niger* ^a)^.

	Normal gravity strain				
Day	Control (mm)^b)^	50 μg/mL (mm)	FIE%	500 μg/mL (mm)	FIE%
2	17.0±1.0	5.7±0.6	65.0±1.7	3.7±0.6	76.1±2.3
5	31.7±1.5	26.0±1.0	19.4±1.5	20.0±1.0	38.0±1.8

^a)^ values given are average of three experiments.

^b)^ the size of the fungal colony is indicated in mm.

FIE%—fungal index efficiency%.

**Table 2:** Biomass of *A*. *niger* after 2 days under normal and microgravity in presence and absence of DMAB-Ch(P) ^a)^.

Fresh Weight			
0 μg/mL of DMAB-Ch(P)		500 μg/mL of DMAB-Ch(P)	
MG (g)	NG (g)	MG (g)	NG (g)
4.97±0.16	4.50±0.05	3.05±0.18	2.51±0.15
Dry Weight			
0.25±0.02	0.17±0.02	0.18±0.01	0.15±0.01

^a)^ values given are average of three experiments.

MG–Microgravity

NG–Normal gravity

*A*. *niger* can adapt well to microgravity conditions without any changes in the chitin metabolism *niger* [8]. Scanning electron microscopic images revealed cell wall shrinkage due to cytoplasmic loss in the presence of DMAB(P)-Ch under normal gravity as well as under microgravity, respectively. Same set of experiment was carried out in the absence of chitosan derivative to rule out the possible artifacts influencing the *A*. *niger* morphology, and none were observed (S2A and S2B Fig). Both spores and mycelium looked healthy in the absence of chitosan derivative under normal gravity as well as under microgravity as shown in S2A and S2B Fig, respectively. The SEM morphological results suggested that the efficacy of DMAB(P)-Ch is not hampered under microgravity conditions.

**Fig 6:**
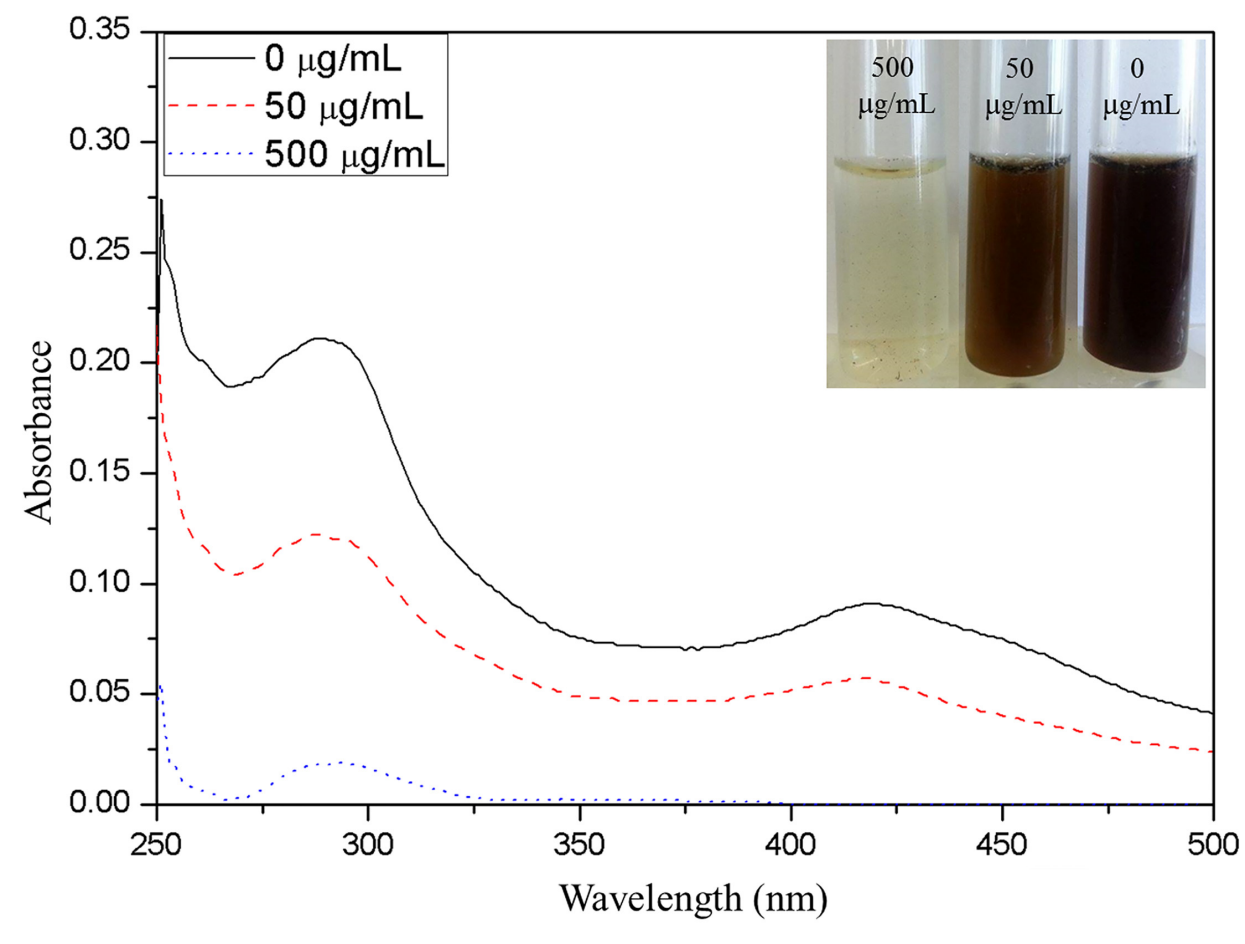
UV-visible spectra of secondary metabolites presence in culture extract both in absence and presence of chitosan derivative. The insets show the color variation of the culture extract by naked-eye.

Generally, the secondary metabolites are synthesized in the cytoplasm and released into the growth medium. The cytoplasmic loss was validated by secondary metabolite profiling by UV-visible spectroscopy (Fig 6) and cyclic voltammetry (S3 Fig). The culture filtrate was analyzed by UV-Visible spectroscopy for qualitative screening of secondary metabolites. Normal profile of secondary metabolites was observed in the absence of chitosan derivative as seen in Fig 6. The bands observed at 254, 280, and 415 nm may be ascertained to the following secondary metabolites and mycotoxins: aurasperone D, yanuthone E, yanuthone D, nigerazine B, and asperenone [27]. In the presence of chitosan derivative all the absorption bands were drastically decreased, especially, the band at 415 nm corresponding to mycelial yellow colorant asperenone completely disappeared at 500 μg/mL of DMAB(P)-Ch. These results correlates well with the naked-eye observation of the culture filtrate as shown in the inset of Fig 6. The decreased concentration of the secondary metabolites could be due to the cytoplasmic loss as observed in SEM (S2 Fig). The suppression of mycotoxins (allergens) by chitosan derivative could be a significant advantage for astronauts in microgravity.

Most of the hyphae appeared to have lost their cytoplasm in the presence of DMAB(P)-Ch (S2 Fig), supporting the hypothesis of chitosan derivative may have permeabilized through the cell wall of *A*. *niger* associated with an increase in reactive oxygen species [28–30], eventually causing cytoplasmic loss. Enhanced performance of DMAB(P)-Ch under microgravity may be due to the changes in the cell membrane electronegativity in response to the free fall condition.

## Conclusions

In the present study, a new chitosan derivative-containing α-aminophosphonate and quaternary ammonium moieties were successfully synthesized by a simple catalyst-free Pudovik reaction. The structure of the chitosan derivative was confirmed by UV-Visible, FT-IR, ^31^P NMR spectroscopies, and by elemental analysis. The fungistatic activity of the chitosan derivative against *A*. *niger* was evaluated under normal gravity and microgravity. A linear relationship was observed between the concentration of chitosan derivative and fungal inhibition efficiency%. Under normal gravity, chitosan derivative exhibited about 76% of inhibition efficiency at the concentration 500 μg/mL for 2 days of incubation. It is noteworthy that the chitosan derivative inhibited about 50% of spore germination under microgravity. The results obtained from UV-visible spectroscopy and cyclic voltammetry for the culture extract suggested that the chitosan derivative might have altered the metabolism of *A*. *niger* for production of secondary metabolites and mycotoxins. A possible application of the synthesized chitosan derivative is that it could be used as a disinfectant in space stations to remove fungal contaminants, which can help to maintain the health of the astronauts as well as the integrity of the spacecraft.

## Supporting Information

S1 Fig Radial hyphal bioassay: Digital photograph of PDA plates containing *A*. *niger* in absence (left) and presence of chitosan derivative. (TIF)

S2 Fig SEM image of *A*. *niger* grown under normal gravity in the absence (A) and presence of chitosan derivative (B). Figure (C) and (D) were SEM image of *A*. *niger* grown microgravity in the absence and presence of chitosan derivative respectively. The insets in Figure (C) and (D) exhibit the morphology of individual spores.
(TIF)

S3 Fig Cyclic voltammetry curves of secondary metabolites from culture extract both in the absence (solid line) and the presence (dotted lines) of different concentrations of chitosan derivative. (TIF)
