# Supplementary figures and images for: Effect of Microgravity on Fungistatic Activity of an α-Aminophosphonate Chitosan Derivative against Aspergillus niger
Source: PLoS One. 2015 Oct 15;10(10):e0139303. doi: 10.1371/journal.pone.0139303 (PMC4607506; doi:10.1371/journal.pone.0139303)

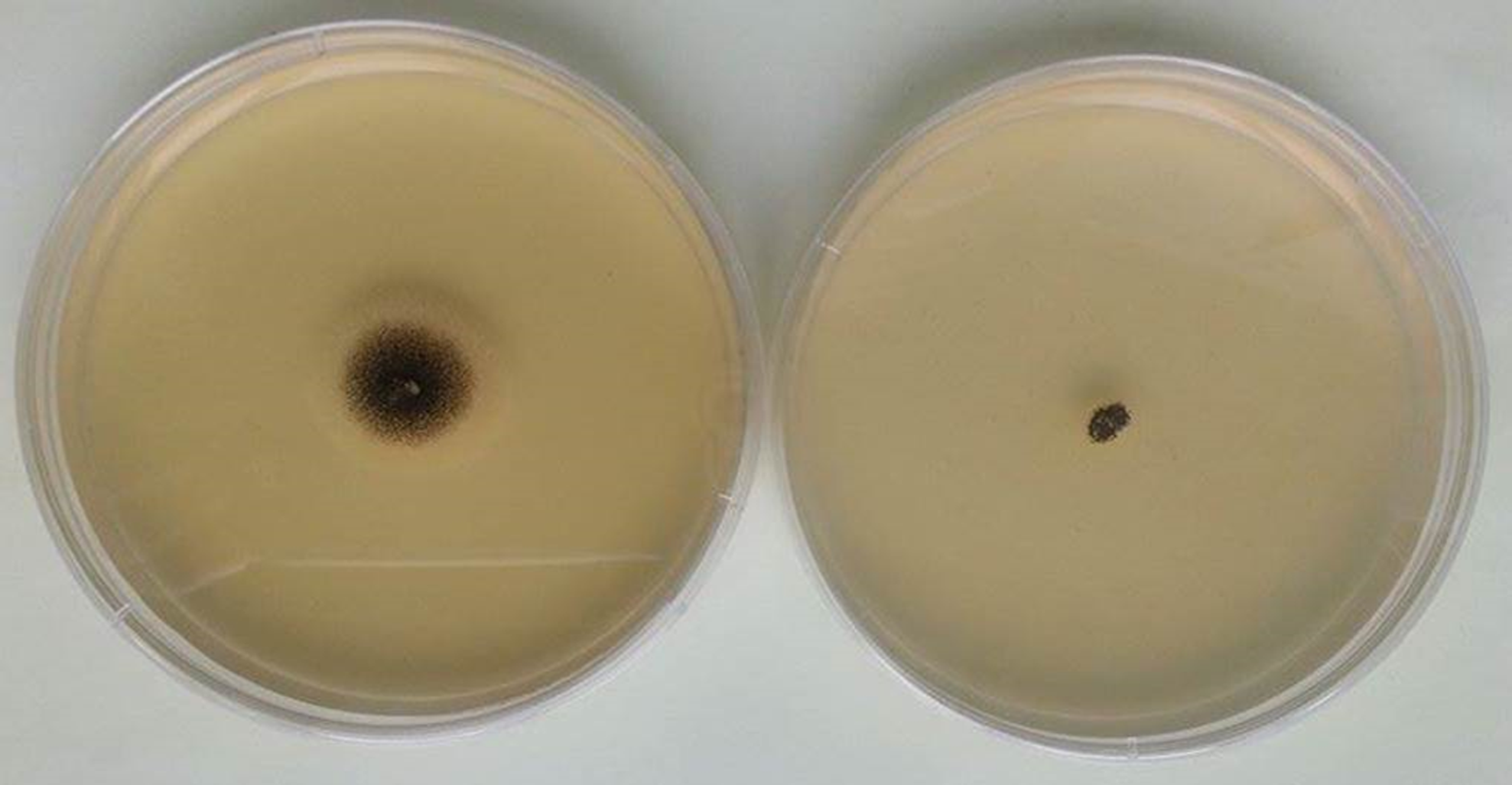

Supplement: S1 Fig — (TIF) [file pone.0139303.s001.tif]

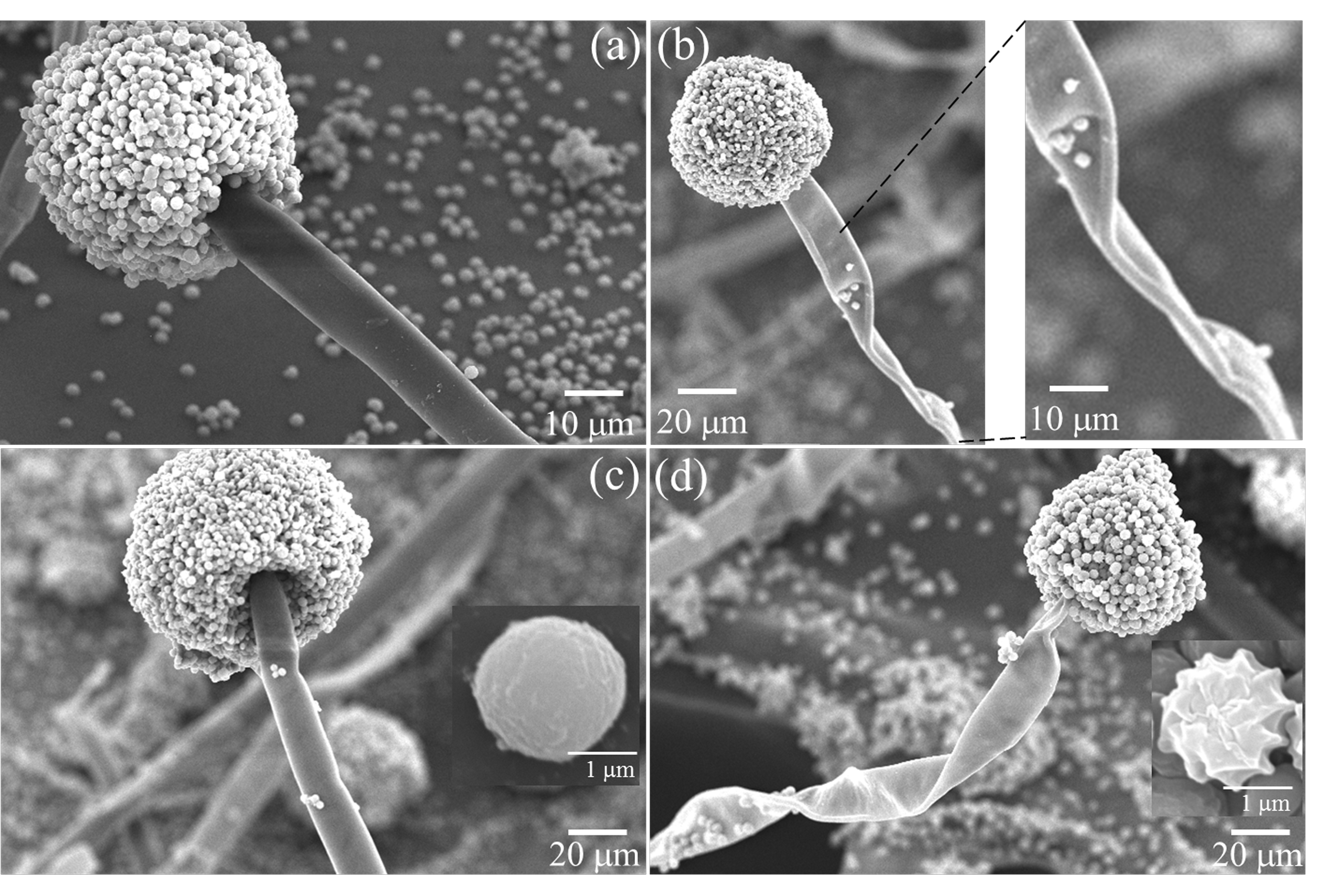

Supplement: S2 Fig — The insets in Figure (C) and (D) exhibit the morphology of individual spores. (TIF) [file pone.0139303.s002.tif]

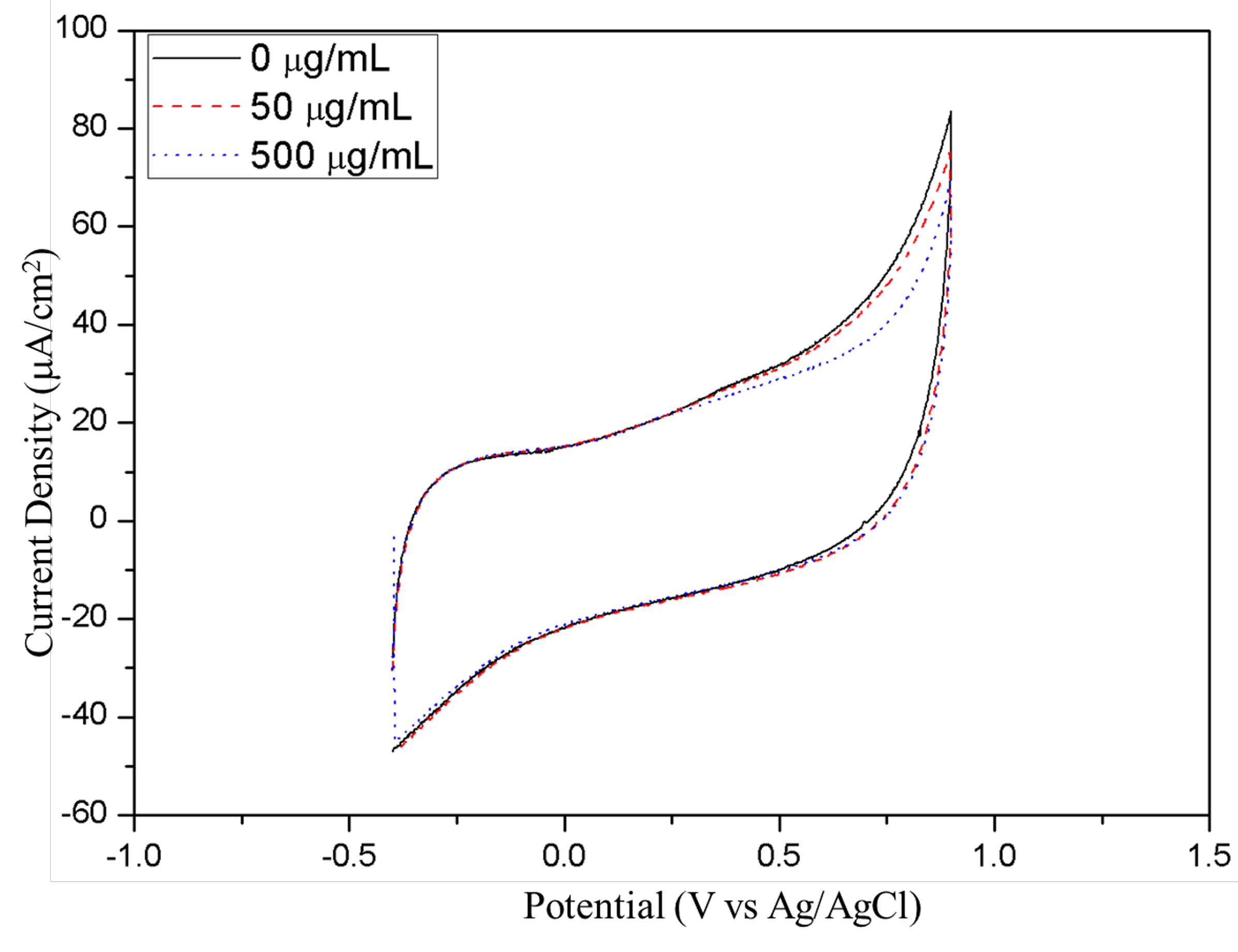

Supplement: S3 Fig — (TIF) [file pone.0139303.s003.tif]
